# Identification of a Novel PDRG1-EZH2-p21 Pathway Controlling Senescence and Tumor Progression in Hepatocellular Carcinoma

**DOI:** 10.7150/ijbs.129113

**Published:** 2026-02-18

**Authors:** Qiang Yang, Lilong Zhang, Wei Li, Zhengle Zhang, Jing Tao, Yuping Rong, Weixing Wang

**Affiliations:** 1 Department of Pancreatic Surgery, Renmin Hospital of Wuhan University, Wuhan, Hubei Province, China.; 2 Department of Hepatobiliary Surgery, Renmin Hospital of Wuhan University, Wuhan, Hubei Province, People's Republic of China.; 3 General Surgery Laboratory, Renmin Hospital of Wuhan University, Wuhan, Hubei, China.

**Keywords:** hepatocellular carcinoma, PDRG1, EZH2, p21, cellular senescence

## Abstract

Hepatocellular carcinoma (HCC) remains a major global health burden with limited therapeutic options and poor prognosis. PDRG1 is upregulated in several malignancies, yet its clinical relevance and mechanistic role in HCC are not fully understood. Here, we investigated the contribution of PDRG1 to HCC progression and delineated the underlying molecular mechanism. Using public datasets, patient specimens, *in vitro* functional assays, and subcutaneous xenograft models, we evaluated PDRG1 expression, biological functions, and downstream pathways. Transcriptome profiling, pathway enrichment analysis, rescue experiments, co-immunoprecipitation, and ChIP-qPCR were performed to define the PDRG1-EZH2-p21 axis. PDRG1 was significantly upregulated in HCC tumor tissues compared with adjacent non-tumor liver tissues and was associated with worse patient survival. Functionally, PDRG1 enhanced HCC cell proliferation, migration, invasion, colony formation, and tumor growth *in vivo*. RNA-seq and enrichment analyses identified cellular senescence as a prominent downstream program regulated by PDRG1. Mechanistically, PDRG1 directly interacted with EZH2, increased H3K27me3 enrichment at the p21 promoter, and suppressed p21 transcription. Restoration of p21 expression attenuated the oncogenic effects of PDRG1, whereas EZH2 overexpression rescued the impaired malignant phenotypes caused by PDRG1 knockdown. Domain-mapping further indicated that the N-terminal residues 36-70 of PDRG1 contribute to its interaction with EZH2. Collectively, our findings identify PDRG1 as a clinically relevant oncogene in HCC and reveal an epigenetic mechanism by which PDRG1 cooperates with EZH2 to repress p21 and bypass senescence. The PDRG1-EZH2-p21 axis may represent a potential biomarker and therapeutic target for HCC.

## Introduction

Liver cancer—most notably hepatocellular carcinoma (HCC), which constitutes the vast majority of diagnosed cases—continue to impose a profound global disease burden and are associated with strikingly poor patient survival 1, 2. Although both regional interventions and systemic therapies have advanced substantially in recent years, many individuals still fail to achieve durable responses and ultimately experience tumor progression and death 3, 4. This clinical reality highlights the urgent need to uncover additional molecular determinants that could reveal actionable therapeutic targets.

PDRG1 was originally described as a transcript modulated by ultraviolet exposure and the p53 signaling pathway 5. Subsequent investigations demonstrated that this gene is consistently elevated in numerous cancer types, including malignancies of the lung, ovary, colon, breast, and esophagus. Functional analyses further indicated that PDRG1 influences several processes central to tumor biology, such as DNA damage responses, resistance to irradiation, cell-cycle transitions, and programmed cell death 6. And PDRG1 promotes the proliferation and migration of glioblastoma multiforme cells by the MEK/ERK/CD44 pathway 7. Experimental depletion of PDRG1 markedly impaired the growth of colon cancer cells 8, and its involvement in radiation tolerance has been documented in both lung carcinoma 9 and nasopharyngeal carcinoma cells 10. Despite these observations, its expression dynamics, pathological relevance, functional roles, and mechanistic contributions in liver cancer remain largely undefined.

Cellular senescence is a durable antiproliferative program initiated by diverse endogenous or environmental insults. This state is chiefly governed by cyclin-dependent kinase inhibitors, including p16 (CDKN2A) and p21 (CDKN1A) 11. Senescent cells commonly display elevated levels of p19, p27, and p53, together with loss of Lamin B1, and characteristically produce a complex secretory profile termed the senescence-associated secretory phenotype (SASP). SASP encompasses inflammatory cytokines, chemokines, growth-promoting signals, and matrix-modifying enzymes that elicit highly context-specific biological outcomes. Senescence can exert tumor-suppressive effects—for example, SASP-derived TNF-α is capable of inducing oxidative stress-driven apoptosis in cancer cells 12 and may propagate senescence to surrounding cells through soluble mediators or direct intercellular communication 13, 14. Conversely, other SASP constituents, such as IL-6, IL-8, CXCL5, and MMP7, have been implicated in fostering malignant initiation, invasiveness, metastatic potential, and immune evasion 15-17.

In the context of HCC, senescence within tumor cells can restrict proliferation and is modulated by metabolic cues and epigenetic regulators, including ammonia and methionine pathways and histone-modifying enzymes 18-20. In contrast, senescence occurring in hepatic stellate cells or hepatocytes has been shown to enhance liver tumorigenesis through SASP-driven inflammatory signaling 21, 22. Collectively, these findings emphasize the dualistic and microenvironment-dependent nature of senescence in liver cancer and underscore the importance of delineating the mechanisms that dictate senescence outcomes during HCC progression. Despite the accumulating evidence that PDRG1 is dysregulated in multiple malignancies, its clinical relevance and mechanistic contribution to HCC remain insufficiently defined. In particular, it is unclear whether PDRG1 directly modulates the senescence program in HCC and, if so, through which upstream epigenetic mechanism PDRG1 controls key senescence effectors such as p21. This unresolved gap limits our understanding of how HCC cells evade durable senescence and thereby sustain malignant progression.

To address this knowledge gap, we systematically investigated the expression pattern and prognostic significance of PDRG1 in HCC cohorts and clinical specimens, and then examined its functional role using gain- and loss-of-function approaches in HCC cell lines and xenograft models. We further combined transcriptome profiling with mechanistic assays and rescue experiments to test the hypothesis that PDRG1 promotes HCC progression by cooperating with EZH2-mediated repression of p21, thereby suppressing cellular senescence and remodeling senescence-associated secretory phenotypes. Conceptually, we identify PDRG1 as an upstream modulator of EZH2-dependent repression of p21 in HCC, providing a mechanistic link between PDRG1 dysregulation and impaired senescence programs that support tumor progression.

## Materials and Methods

### Cell culture

HuH-7, SNU449, HLF, and 293T cells were sourced from the Cell Bank of the Chinese Academy of Sciences (Shanghai, China) and maintained in DMEM (Hyclone, Logan, USA) supplemented with 10% fetal bovine serum (QmSuero, China) under standard culture conditions (37 °C, 5% CO₂, humidified).

### Stable cell line generation

Lentiviruses were produced by co-transfecting 293T cells with the expression plasmids and the packaging plasmids (psPAX2 and pMD2.G). The supernatants were collected after 48 h and filtered through 0.45 μm filters (Millipore, Temecula, CA, USA), then concentrated using Amicon Ultra centrifugal filters (Millipore 100 KD MWCO). The concentrated viruses were used to infect HuH-7, SNU449 and HLF cells. Stable transfection cell lines were selected with 2 ug/ml puromycin for 5 days. Stable overexpression or knockdown of PDRG1 and EZH2 cells were generated by lentivirus infection.

### Cell proliferation and colony formation

Cell proliferation was assessed using CCK-8 and EdU assays. For colony formation assay, 1 × 10^3^ cells were cultured in 6-well plate at 37 °C for 14 days. Visible colonies were washed twice with phosphate-buffered saline, fixed and stained with 4% paraformaldehyde and crystal violet, respectively. The number of colonies was counted visually.

### Migration and invasion assays

For wound-healing assays, confluent monolayers were scratched and cultured in serum-free medium. Images were captured at 0 and 48 hours. For Transwell assays, cells were plated into upper chambers (with or without Matrigel). Migrated or invaded cells were fixed, stained, and counted.

### Bioinformatics analyses

Using the HCDDB database to analyze the expression of PDRG1 in different datasets of liver cancer patients. Publicly available transcriptomic datasets, including TCGA-LIHC and GEO datasets were retrieved from the UCSC Xena and GEO databases. Survival analyses including overall survival (OS), disease-free survival (DFS), disease specific survival (DSS), and progression-free survival (PFS) were performed using Cox proportional hazards models via the survival (v3.2) and survminer (v0.4.9) packages.

### RNA extraction, reverse transcription and qRT-PCR

These relevant protocols were carried out as previously described 23. The cDNA Synthesis SuperMix and Top Green qPCR SuperMix were purchased from TransGen Biotech Company (AT311-03; AQ131-03).

### RNA-seq and data processing

Total RNA from PDRG1-overexpressing and control HCC cells was extracted using RNAiso Plus. RNA quality was evaluated with Agilent 2100 Bioanalyzer. Poly(A)+ mRNA was enriched, fragmented, reverse-transcribed, and subjected to library construction using the Illumina TruSeq Kit. Sequencing was performed on an Illumina NovaSeq 6000 platform. Raw RNA-seq reads were subjected to quality control, and adapters/low-quality bases were removed prior to alignment. Clean reads were aligned to the human reference genome (GRCh38/hg38) using STAR. Gene-level read counts were quantified using featureCounts based on gene annotation, and differential expression analysis was performed using DESeq2. Genes with an absolute log2 (fold change) ≥ 0.585 and a false discovery rate (FDR) < 0.05 (Benjamini-Hochberg adjusted *P* value) were considered significantly differentially expressed. Unless otherwise stated, statistical tests were two-sided and adjusted *P* values are reported for RNA-seq comparisons. Heatmaps and volcano plots were generated using pheatmap (v1.0.12) and ggplot2 (v3.3.3), respectively. Gene set enrichment analysis (GSEA) was conducted using the clusterProfiler R package (v4.0.5). Genes were ranked by log2 (fold change) from DESeq2, and enrichment was evaluated against the MSigDB Hallmark gene set collection. Gene sets with FDR < 0.05 were considered significantly enriched. Enrichment results were summarized using normalized enrichment scores and visualized for representative pathways.

### Western blot analysis

Cells or tissues were lysed using RIPA buffer (Servicebio, China, G2002) supplemented with protease inhibitor (Servicebio, China, G2008) to obtain total protein extracts. Samples were resolved by 8-12% SDS-PAGE and electrotransferred onto PVDF membranes. After blocking, the membranes were probed overnight at 4 °C with the designated primary antibodies, incubated with secondary antibodies, and visualized via Bio-Rad ChemiDoc MP imaging. Signal intensities were quantified with ImageLab software, with GAPDH used as a normalization control. Antibodies against DDDDK/Flag (Proteintech, China, 20543-1-AP), HA (Proteintech, China, 51064-2-AP), PDRG1 (Proteintech, China, 16968-1-AP), E-cadherin (Proteintech, China, 20874-1-AP), N-cadherin (Proteintech, China, 22018-1-AP), Vimentin (Proteintech, China, 10366-1-AP), Beta Catenin (Proteintech, China, 51067-2-AP), p21(Proteintech, China, 28248-1-AP), RB (Proteintech, China, 10048-2-Ig), Phospho-RB (Proteintech, China, 84692-1-RR), IL-1 beta (Proteintech, China, 26048-1-AP), IL-6 (Proteintech, China, 21865-1-AP), IL-8 (Proteintech, China, 27095-1-AP) and EZH2 (Proteintech, China, 21800-1-AP) were purchased from Proteintech Technology. Antibodies against p-CDK2 (Abcam, America, EP2234Y) was purchased from Abcam Technology.

### Gene expression correlation analysis using GEPIA2

Gene expression correlation analyses were performed using the Gene Expression Profiling Interactive Analysis 2 (GEPIA2) web server (http://gepia2.cancer-pku.cn/#index). Correlations between the indicated genes were evaluated within the LIHC tumor cohort using the “Correlation Analysis” function in the “Expression Analysis” module. Pearson's rank correlation was applied, and all analyses were conducted using the default parameters implemented by GEPIA2, with tumor samples only (LIHC Tumor).

### Immunohistochemistry (IHC)

Paraffin-embedded subcutaneous tumor tissue, and human HCC and adjacent liver tissues were sectioned at 4 μm. After deparaffinization and rehydration, antigen retrieval was performed using citrate buffer (pH 6.0), depending on the primary antibody. Endogenous peroxidase activity was quenched using 3% H₂O₂. Sections were incubated with primary antibodies against PDRG1, EZH2, p21, Ki-67, IL-6, IL-1β, p-RB, and p-CDK2 at 4 °C overnight, followed by HRP-conjugated secondary antibodies. Staining was visualized using DAB and counterstained with hematoxylin. Quantification of IHC signals was performed using ImageJ. Clinicopathological correlations were assessed based on IHC scoring.

### Co-immunoprecipitation (Co-IP) and mass spectrometry (IP-MS)

Cells were lysed with NP-40 lysis buffer supplemented with protease inhibitors. Lysates were incubated with anti-PDRG1 antibody overnight, followed by protein A/G beads. Co-precipitated proteins were eluted, separated on SDS-PAGE, and stained with silver stain. For IP-MS, gel slices were digested with trypsin, and peptides were analyzed on a Thermo Scientific Orbitrap Exploris 480 mass spectrometer. Interacting proteins were identified using MaxQuant with an FDR < 1%.

### Chromatin immunoprecipitation (ChIP)-qPCR

ChIP assays were performed using the Beyotime ChIP kit according to standard protocols. Briefly, cells were cross-linked with 1% formaldehyde, lysed, and sonicated to obtain 200-500 bp chromatin fragments. Immunoprecipitation was performed using anti-EZH2, anti-H3K27me3, or control IgG. Purified DNA was analyzed by qPCR using primers targeting the p21 promoter.

### Construction of truncation mutants

Flag-tagged EZH2 truncation mutants and HA-tagged PDRG1 truncations were generated by PCR-based cloning using high-fidelity polymerase. Fragments were cloned into pcDNA3.1 vectors using ClonExpress II One-Step Cloning Kit (Vazyme). Correct insertion and frame fidelity were verified by Sanger sequencing. Plasmids were transfected into SNU449 cells using Lipofectamine 3000.

### Molecular docking and structural modeling

Protein structural models for PDRG1 and EZH2 were obtained from the AlphaFold Protein Structure Database. Protein-protein docking simulations were carried out using the HDOCK server. The top-scoring models were refined based on binding energy and interface complementarity. Protein interaction surfaces and residues contributing to the interaction were visualized using PyMOL (v2.5).

### Animal studies

All animal experiments were performed in accordance with institutional guidelines for animal care and were conducted under the approval of Renmin Hospital of Wuhan University. Four-week-old male BALB/c-nu mice (Shulaibao, Wuhan, China) were housed in specific pathogen-free (SPF) facilities with ad libitum access to food and water. Tumor cells were subcutaneously injected into the flanks of 4-week-old BALB/c nude mice. Tumor growth was monitored every 3 days. Tumor length (L) and width (W) were measured using digital calipers by an investigator blinded to group allocation, and tumor volume was calculated using the formula V = 0.5 × L × W². Body weight and general health status were monitored in parallel. Mice were anesthetized for procedures when required using isoflurane inhalation, and analgesia was provided as indicated by institutional guidelines to minimize discomfort. At the study endpoint, mice were euthanized by CO₂ inhalation, followed by cervical dislocation as a secondary physical method to confirm death, tumors were excised, photographed, and processed for IHC staining.

### Statistical analysis

R Project and RStudio were used for sequencing analysis, while GraphPad Prism 7.0 and SPSS 16.0 were used for statistical analysis.

Where appropriate, data are expressed as mean ± standard error of the mean (SEM). For intergroup comparisons, a two-tailed Student's t-test (parametric, normal distribution) or Welch's t-test (unequal variances) was used. For non-normally distributed data, the Wilcoxon rank-sum test was applied. Chi-square or Fisher's exact tests were used for categorical data. All *in vitro* experiments were repeated for a total of at least 3 times. *p*-values of less than 0.05 were considered significant.

## Results

### PDRG1 is overexpressed in HCC and correlates with adverse clinicopathological features

**Figure 1:**
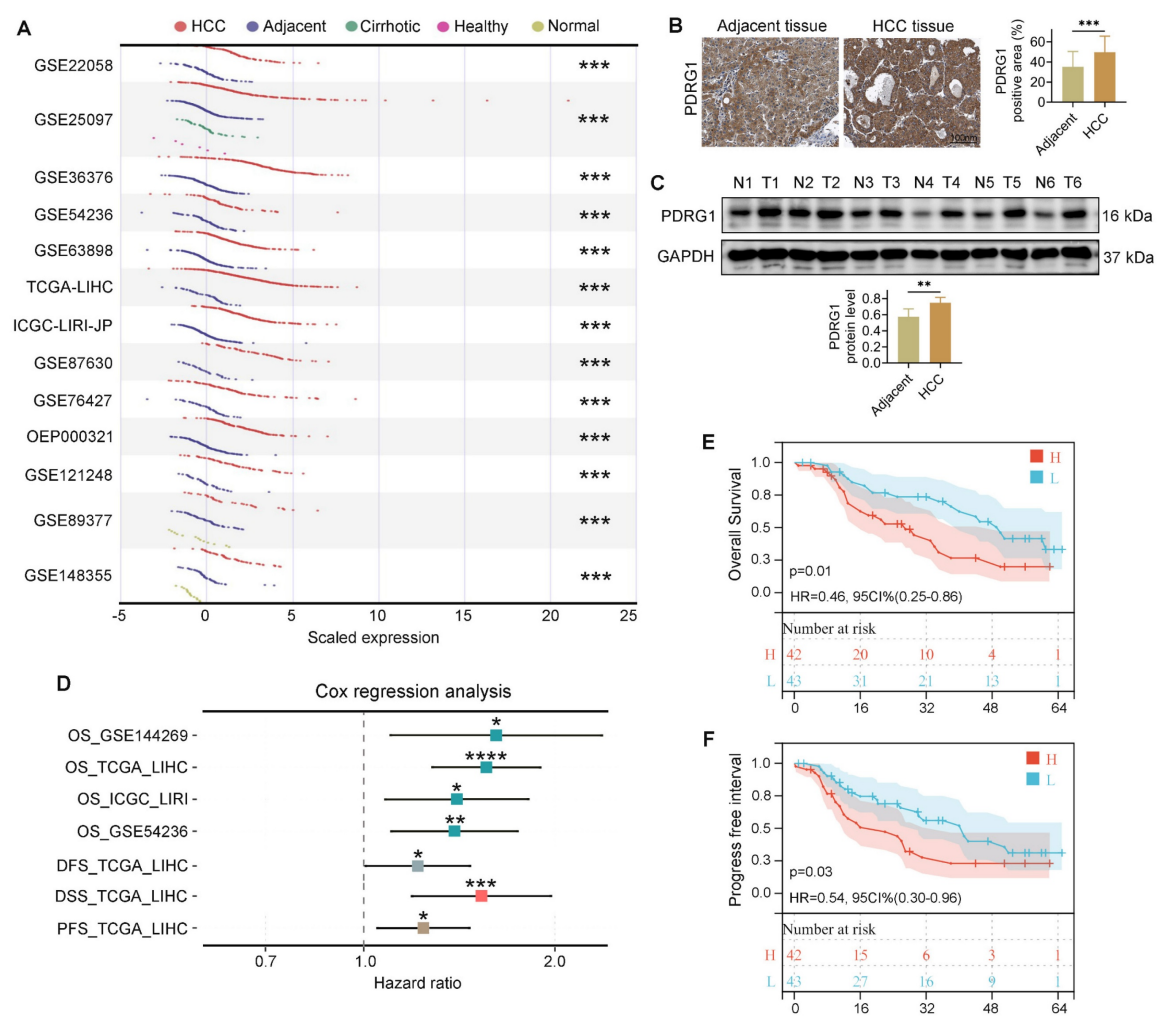
PDRG1 is upregulated in HCC and associated with unfavorable prognosis. (A) Expression analysis of PDRG1 across multiple HCC datasets from the HCCDB database showing significant upregulation in tumor tissues compared with non-tumorous liver tissues. (B, C) Representative immunoblotting and IHC images of PDRG1 expression in paired HCC and adjacent noncancerous tissues from our cohort (n = 86). (D) The Cox regression analysis showed that in the public dataset, the higher the expression level of PDRG1, the worse the overall survival (OS), disease-free survival (DFS), disease specific survival (DSS), and progression-free survival (PFS). (E, F) OS and PFS analyses in our clinical cohort confirming that high PDRG1 expression predicts poor prognosis. *p < 0.05, **p < 0.01, ***p < 0.001.

To investigate the involvement of PDRG1 in HCC, we first analyzed multiple HCC datasets from the HCCDB database 24 and observed consistent upregulation of PDRG1 in tumor tissues compared with non-tumorous liver tissues (Fig. 1A).

Consistent with the bioinformatic findings, both WB and IHC performed on clinical samples from our center revealed markedly increased PDRG1 expression in HCC specimens compared with adjacent noncancerous tissues (Fig. 1B, 1C; patient information in Table S1). Furthermore, Cox regression analyses across several HCC cohorts showed that high PDRG1 expression was significantly associated with poorer OS, DFS, DSS, and PFS (Fig. 1D). And multivariate Cox regression analysis using TCGA-LIHC clinical variables further demonstrated that PDRG1 expression independently predicts unfavorable OS and PFS in HCC (Table S2, S3). These results were corroborated by survival data from our clinical cohort (Fig. 1E, 1F).

Collectively, these findings demonstrate that PDRG1 is significantly upregulated in HCC tumor tissues compared with adjacent non-tumor liver tissues and is associated with more aggressive disease features and poorer prognosis.

### Modulation of PDRG1 expression regulates the tumorigenic properties of HCC cells

**Figure 2:**
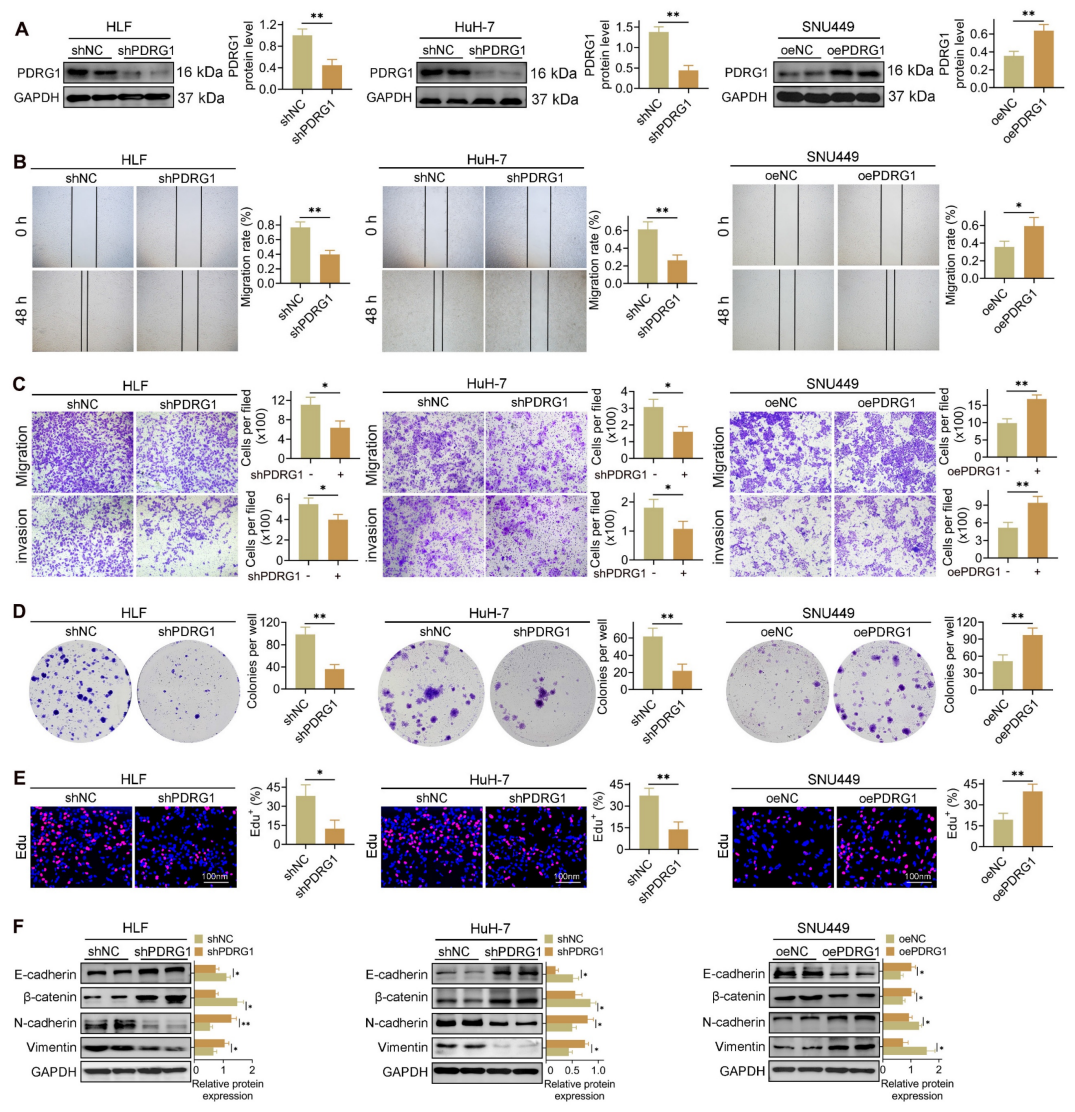
PDRG1 regulates proliferation, migration, invasion, and EMT in HCC cells. (A) Western blot validation of PDRG1 knockdown efficiency in HuH-7 and HLF cells and overexpression in SNU449 cells. (B) Wound-healing assays showing impaired migration upon PDRG1 knockdown and enhanced migration upon PDRG1 overexpression. (C) Transwell migration and invasion assays demonstrating reduced motility and invasiveness after PDRG1 knockdown and increased motility after PDRG1 overexpression. (D) Colony formation assays showing decreased clonogenicity upon PDRG1 knockdown and increased colony-forming ability with PDRG1 overexpression. (E) EdU incorporation assays indicating decreased proliferation in PDRG1-silenced cells and elevated proliferation in PDRG1-overexpressing cells. (F) Western blot analysis of EMT markers showing increased epithelial markers (E-cadherin and β-catenin) and decreased mesenchymal markers (N-cadherin and Vimentin) after PDRG1 knockdown, with the opposite effect upon PDRG1 overexpression. All experiments were performed with three independent biological replicates (n = 3). *p < 0.05, **p < 0.01, ***p < 0.001.

To determine the functional role of PDRG1 in HCC, we established PDRG1-knockdown HuH-7 and HLF cell lines, as well as PDRG1-overexpressing SNU449 cells. WB confirmed efficient knockdown and overexpression (Fig. 2A). Wound-healing assays showed that PDRG1 knockdown markedly impaired migratory ability, whereas PDRG1 overexpression enhanced migration (Fig. 2B). Similarly, Transwell assays demonstrated reduced migration and invasion upon PDRG1 silencing and increased invasive capacity upon PDRG1 overexpression (Fig. 2C).

PDRG1 depletion significantly inhibited colony formation and EdU incorporation in HuH-7 and HLF cells, whereas PDRG1 overexpression markedly promoted proliferation in SNU449 cells (Fig. 2D, 2E). These results were further supported by CCK-8 assays (Fig. S1A-C), which consistently showed reduced viability in PDRG1-depleted cells and enhanced proliferation in PDRG1-overexpressing cells. WB analysis of epithelial-mesenchymal transition (EMT) markers revealed that PDRG1 knockdown increased epithelial markers (E-cadherin and β-catenin) and decreased mesenchymal markers (N-cadherin and Vimentin), while PDRG1 overexpression induced the opposite changes (Fig. 2F).

Overall, these results indicate that PDRG1 promotes proliferation, migration, invasion, and EMT in HCC cells, supporting its role as a functional driver of HCC progression.

### PDRG1 drives HCC progression by suppressing tumor cell senescence

**Figure 3:**
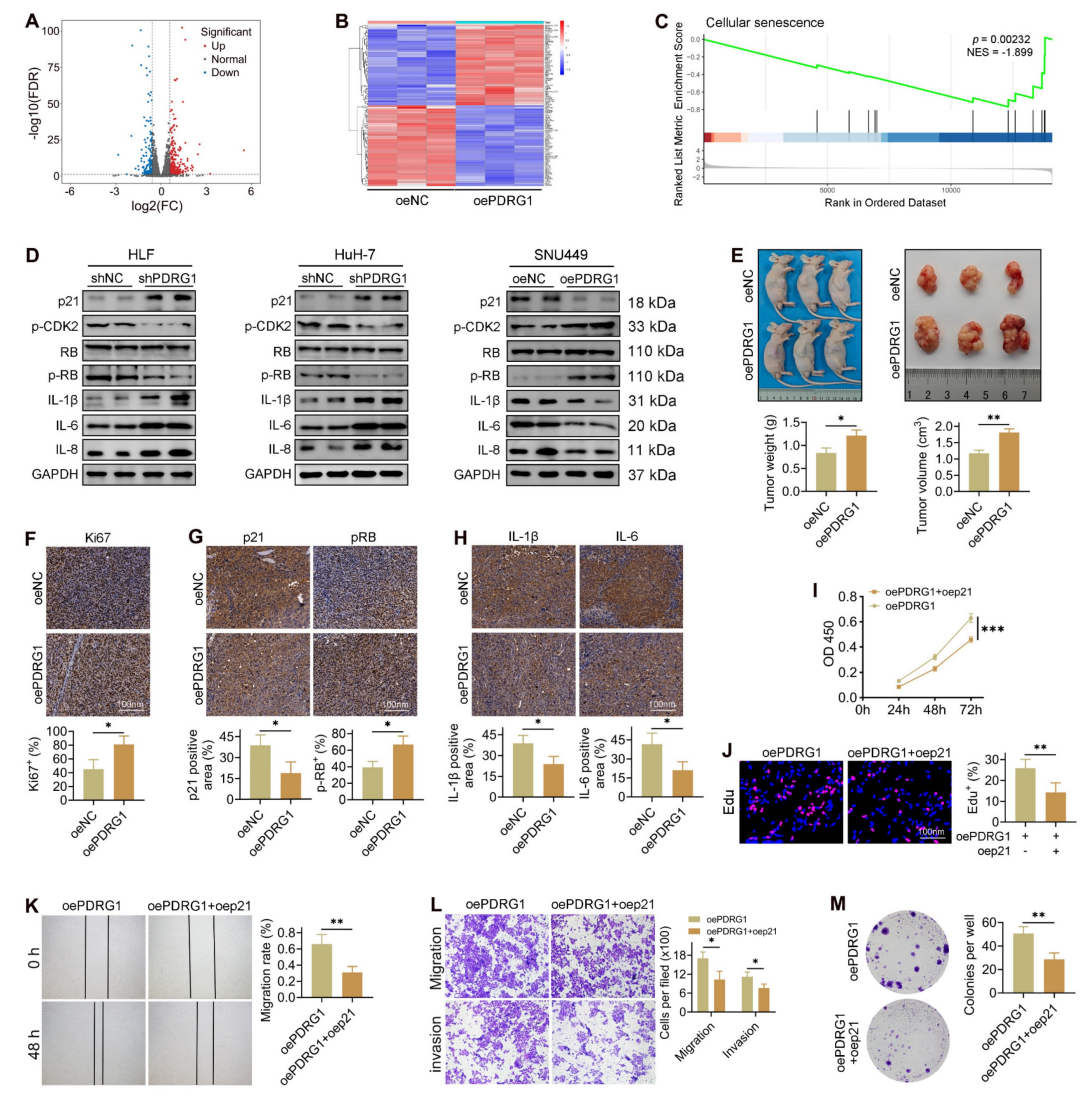
Transcriptomic profiling reveals that PDRG1 suppresses tumor cell senescence and promotes HCC progression. (A) Volcano plot showing 696 differentially expressed genes upon PDRG1 overexpression. (B) Heatmap of the top significantly altered genes in PDRG1-overexpressing cells. (C) GSEA showing enrichment of senescence-related signatures in PDRG1-overexpressing cells. (D) Western blot analysis showing that PDRG1 knockout increases p21 and SASP factors (IL-6, IL-8, and IL-1β) while decreasing p-CDK2 and p-RB; conversely, PDRG1 overexpression reduces p21 and SASP factors and increases p-CDK2 and p-RB. (E) Xenograft assays show that SNU449 cells overexpressing PDRG1 display enhanced tumor growth, as evidenced by increased tumor volume and tumor weight. (F-H) IHC analysis of Ki-67, p-RB, p21, IL-6, and IL-1β in xenograft tumors showing reduced senescence-associated markers in PDRG1-overexpressing tumors. (I) CCK-8 assays showing that p21 restoration significantly attenuates proliferation in PDRG1-overexpressing SNU449 cells. (J) EdU incorporation assays showing that p21 restoration reduces DNA synthesis in PDRG1-overexpressing SNU449 cells. (K) Wound-healing assays showing that p21 overexpression reverses the enhanced migratory capacity induced by PDRG1 overexpression. (L) Transwell migration and invasion assays showing that p21 restoration suppresses PDRG1-induced increases in migration and invasion. (M) Colony formation assays showing that p21 overexpression markedly reduces clonogenic growth in PDRG1-overexpressing SNU449 cells. All experiments were performed with three independent biological replicates (n = 3). *p < 0.05, **p < 0.01, ***p < 0.001.

To elucidate how PDRG1 promotes HCC progression, we performed transcriptome sequencing in PDRG1-overexpressing cells. A total of 696 differentially expressed genes (89 downregulated, 607 upregulated) were identified (Fig. 3A), and the heatmap highlights the most significantly altered genes (Fig. 3B). The GSEA results showed that in SNU449 cells with overexpression of the PDRG1 gene, the senescence pathways were significantly inhibited, indicating that PDRG1 may play a role in inhibiting senescence (Fig. 3C).

Consistent with this, silencing PDRG1 led to a pronounced increase in p21 expression, accompanied by elevated levels of SASP-related cytokines, including IL-6, IL-8, and IL-1β (Fig. 3D, Fig. S2). Additionally, phosphorylation of CDK2 and RB was significantly reduced in PDRG1-silenced cells, whereas total RB levels remained unchanged (Fig. 3D), indicating disruption of the CDK2-RB signaling axis. Conversely, PDRG1 overexpression produced the opposite pattern, suppressing p21 and SASP-associated cytokines while restoring CDK2 and RB phosphorylation without affecting total RB abundance (Fig. 3D). To validate these findings, SNU449 cells stably overexpressing PDRG1 were subcutaneously implanted into nude mice. Tumors derived from PDRG1-overexpressing cells exhibited significantly accelerated growth (Fig. 3E). IHC analysis revealed increased Ki-67 and phosphorylated RB and decreased p21, IL-6, and IL-1β expression in PDRG1-overexpressing tumors (Fig. 3F-H), further supporting a role for PDRG1 in suppressing tumor cell senescence.

Together, these findings indicate that PDRG1 promotes HCC progression at least in part by inhibiting senescence-related signaling pathways.

### Restoration of p21 expression abrogates the oncogenic effects of PDRG1

To determine whether p21 mediates the pro-tumorigenic function of PDRG1, we restored p21 expression in PDRG1-overexpressing cells. CCK-8 assays demonstrated that p21 reintroduction significantly attenuated the enhanced proliferation induced by PDRG1 overexpression (Fig. 3I). EdU incorporation assays further confirmed that p21 markedly reduced DNA synthesis activity in PDRG1-overexpressing cells (Fig. 3J).

In functional assays, recovery of p21 expression suppressed the PDRG1-induced increase in cell migration, as shown by wound-healing assays (Fig. 3K). Transwell migration and invasion assays yielded similar results, with p21 effectively reversing the promigratory and pro-invasive phenotypes induced by PDRG1 (Fig. 3L). Additionally, colony formation assays revealed that p21 significantly diminished the clonogenic capacity of PDRG1-overexpressing cells (Fig. 3M).

Together, these findings demonstrate that PDRG1 promotes proliferation, migration, invasion, and tumorigenicity by suppressing p21-mediated cellular senescence, establishing the PDRG1-p21-senescence axis as a key regulatory pathway in HCC progression.

### PDRG1 interacts with EZH2 and positively regulates its expression in HCC

To delineate the molecular machinery through which PDRG1 drives HCC progression, we conducted IP-MS to identify PDRG1-associated proteins. This unbiased proteomic screening revealed a cluster of chromatin-regulatory factors enriched in the PDRG1 interactome, among which the histone methyltransferase EZH2 ranked prominently. Given EZH2's established role as the catalytic core of the Polycomb Repressive Complex 2 (PRC2) complex 25, 26 and its well-documented involvement in epigenetic silencing during HCC progression 27, 28, we prioritized EZH2 for further validation.

**Figure 4:**
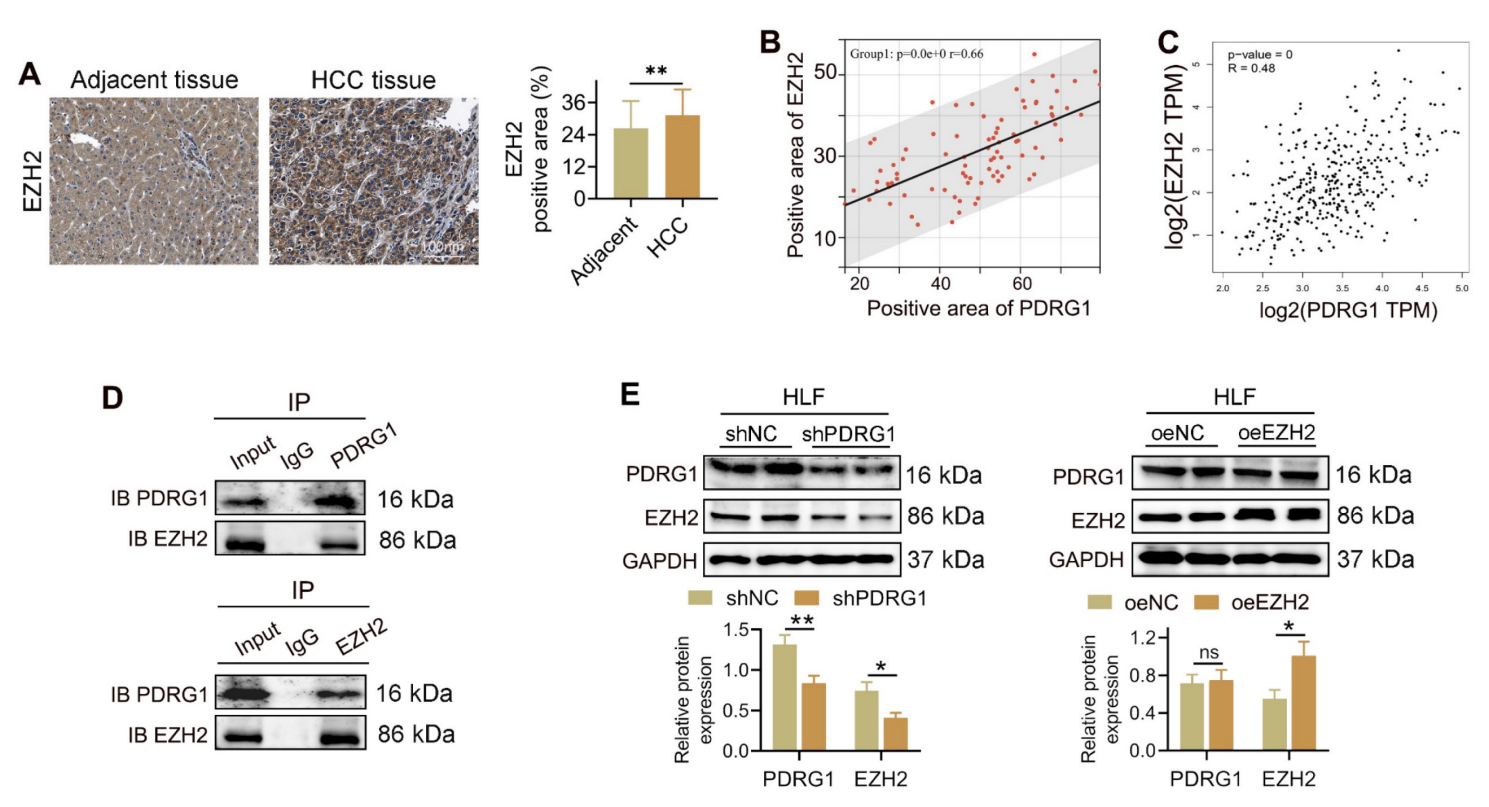
PDRG1 interacts with EZH2 and positively regulates EZH2 expression in HCC. (A) IHC staining showing elevated EZH2 expression in HCC tissues compared with adjacent non-tumor tissues (n = 86). (B) Correlation analysis of PDRG1 and EZH2 protein expression in clinical HCC samples. (C) TCGA-LIHC dataset confirming the positive correlation between PDRG1 and EZH2 mRNA expression. (D) Co-IP assays showing direct binding between PDRG1 and EZH2 in HLF cells. (E) Western blot showing that PDRG1 knockdown reduces EZH2 protein levels, whereas EZH2 overexpression does not affect PDRG1 expression. All experiments were performed with three independent biological replicates (n = 3). *p < 0.05, **p < 0.01, ***p < 0.001.

IHC revealed that EZH2 expression was markedly elevated in HCC tissues compared to adjacent non-tumor tissues (Fig. 4A). Quantitative analysis demonstrated a significant positive correlation between the expression levels of PDRG1 and EZH2 in clinical HCC samples (Fig. 4B), which was further confirmed using TCGA-LIHC transcriptome data (Fig. 4C).

To determine whether PDRG1 physically interacts with EZH2, we performed CO-IP assays. PDRG1 antibody successfully pulled down EZH2, and reciprocally, EZH2 antibody precipitated PDRG1, demonstrating a direct interaction between the two proteins (Fig. 4D). Functionally, knockdown of PDRG1 resulted in a pronounced reduction of EZH2 protein levels in HLF cells, whereas overexpression of EZH2 did not alter PDRG1 expression (Fig. 4E). These findings suggest that PDRG1 positively regulates EZH2 abundance rather than the reverse.

Collectively, these results indicate that PDRG1 interacts with EZH2, providing a mechanistic link between PDRG1 overexpression and the enhanced epigenetic repression observed in HCC.

### EZH2 restores the suppressed malignant phenotypes caused by PDRG1 knockdown

**Figure 5:**
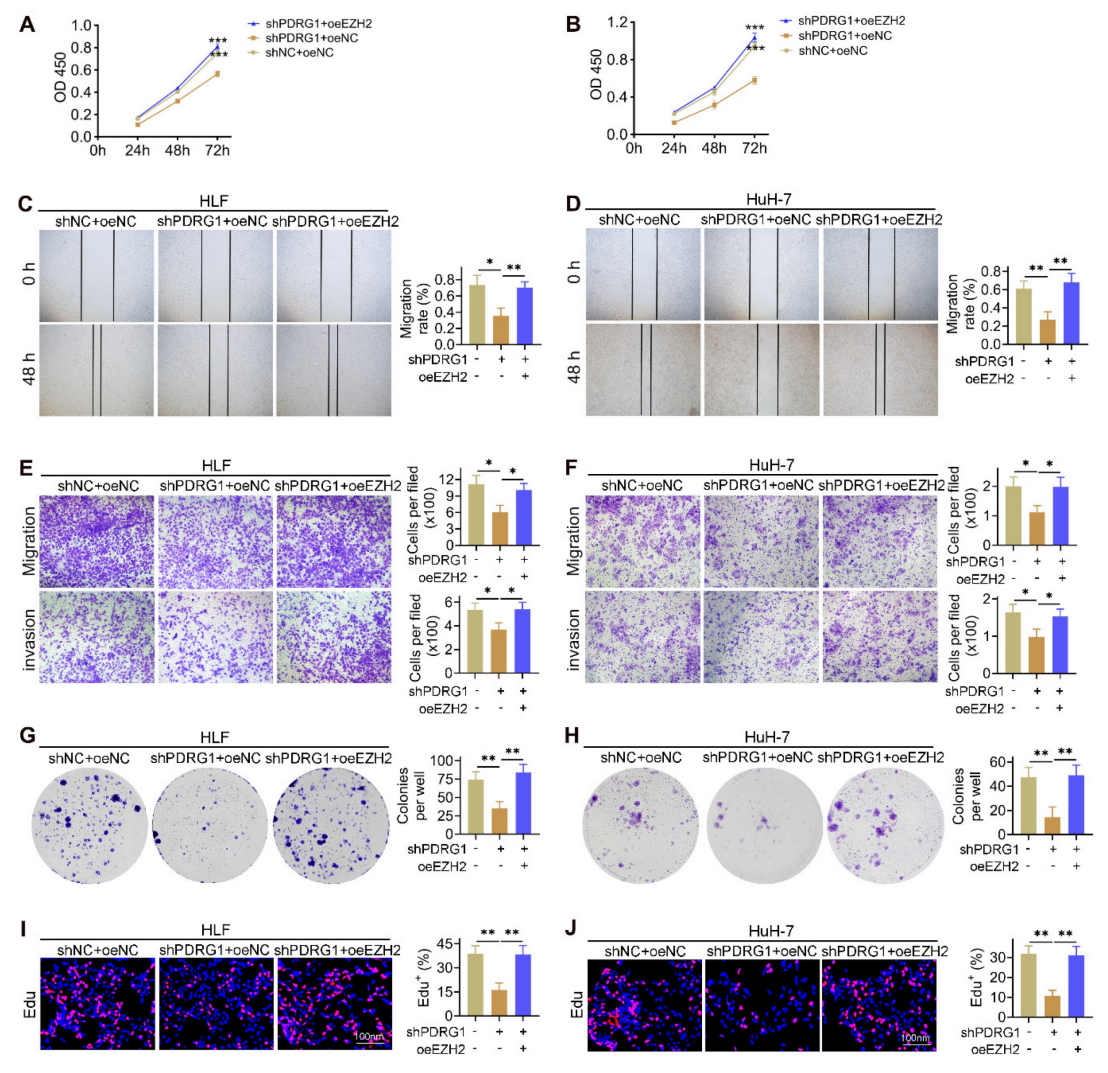
EZH2 restores malignant phenotypes suppressed by PDRG1 knockdown. (A, B) CCK-8 assays showing that EZH2 overexpression restores cell proliferation in PDRG1-silenced HLF and HuH-7 cells. (C, D) Wound-healing assays showing that EZH2 overexpression rescues the migration defects induced by PDRG1 knockdown. (E, F) Transwell assays demonstrating that EZH2 overexpression reverses PDRG1 knockdown-mediated suppression of migration and invasion. (G, H) Colony formation assays showing increased clonogenicity upon EZH2 overexpression in PDRG1-silenced cells. (I, J) EdU assays showing that EZH2 overexpression restores reduced DNA synthesis in PDRG1-knockdown cells. All experiments were performed with three independent biological replicates (n = 3). *p < 0.05, **p < 0.01, ***p < 0.001.

To determine whether EZH2 mediates the oncogenic effects of PDRG1, we restored EZH2 expression in PDRG1-silenced HCC cells. CCK-8 assays showed that knockdown of PDRG1 markedly suppressed cell proliferation, whereas EZH2 overexpression significantly rescued this inhibitory effect in both HLF and HuH-7 cells (Fig. 5A, 5B). Wound-healing assays demonstrated that PDRG1 knockdown substantially impaired cell migratory capacity, while EZH2 restoration effectively reversed this phenotype (Fig. 5C, 5D). Consistently, Transwell migration and invasion assays confirmed that EZH2 overexpression restored the migratory and invasive abilities suppressed by PDRG1 knockdown (Fig. 5E, 5F). Colony formation assays further revealed that PDRG1 depletion significantly reduced clonogenic potential, whereas reintroduction of EZH2 markedly increased colony-forming ability (Fig. 5G, 5H). In addition, EdU assays showed decreased DNA synthesis following PDRG1 knockdown, which was robustly rescued upon EZH2 overexpression (Fig. 5I, 5J).

Together, these results demonstrate that EZH2 is a critical downstream effector of PDRG1, and the PDRG1-EZH2 axis is essential for sustaining the proliferative, migratory, invasive, and clonogenic capabilities of HCC cells.

### PDRG1 regulates p21 transcription through EZH2-mediated H3K27me3 modification

**Figure 6:**
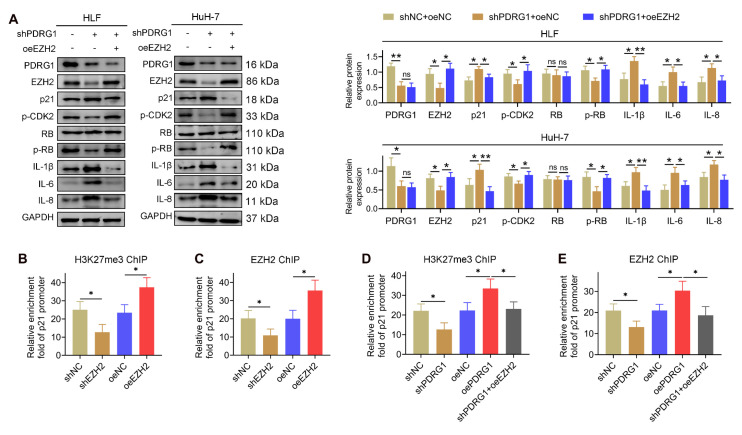
PDRG1 regulates p21 transcription through EZH2-mediated H3K27me3 modification. (A) Western blot analysis showing that PDRG1 knockdown decreases EZH2, p-CDK2, and p-RB levels while increasing p21 and SASP factors (IL-1β, IL-6, and IL-8); ectopic EZH2 expression reverses these changes. (B, C) ChIP-qPCR analysis showing that EZH2 knockdown reduces, whereas EZH2 overexpression increases, EZH2 occupancy and H3K27me3 enrichment at the p21 promoter in HLF cells. (D, E) ChIP-qPCR analysis demonstrating that PDRG1 knockdown decreases EZH2 recruitment and H3K27me3 levels at the p21 promoter, both of which are restored by EZH2 re-expression in HLF cells. All experiments were performed with three independent biological replicates (n = 3). *p < 0.05, **p < 0.01.

To further investigate how the PDRG1-EZH2 axis regulates cellular senescence, we examined changes in key senescence-related proteins. Western blotting revealed that PDRG1 knockdown led to decreased EZH2 and p-CDK2 levels, reduced phosphorylation of RB, and marked upregulation of p21 and the SASP factors IL-1β, IL-6, and IL-8 in both HLF and HuH-7 cells (Fig. 6A). Importantly, restoration of EZH2 expression reversed these effects, reducing p21 and inflammatory cytokines while restoring p-CDK2 and p-RB levels. These findings indicate that EZH2 mediates the regulatory effects of PDRG1 on cell-cycle signaling and SASP expression.

Given that EZH2 is the catalytic subunit of PRC2 responsible for H3K27 trimethylation, we next explored whether PDRG1 influences EZH2-mediated transcriptional silencing of p21. ChIP-qPCR analysis showed that EZH2 knockdown significantly decreased H3K27me3 enrichment at the p21 promoter, whereas EZH2 overexpression markedly increased H3K27me3 occupancy (Fig. 6B). Similarly, EZH2 enrichment at the p21 promoter was reduced upon EZH2 knockdown and enhanced following EZH2 overexpression (Fig. 6C).

Consistent with these results, PDRG1 knockdown reduced H3K27me3 and EZH2 enrichment at the p21 promoter, while restoring EZH2 expression rescued both modifications (Fig. 6D, 6E). These findings collectively demonstrate that PDRG1 promotes EZH2-dependent H3K27 trimethylation at the p21 promoter, thereby suppressing p21 transcription and attenuating senescence-associated signaling.

### Structural identification of PDRG1-EZH2 binding interfaces and functional requirement of their interaction in HCC progression

**Figure 7:**
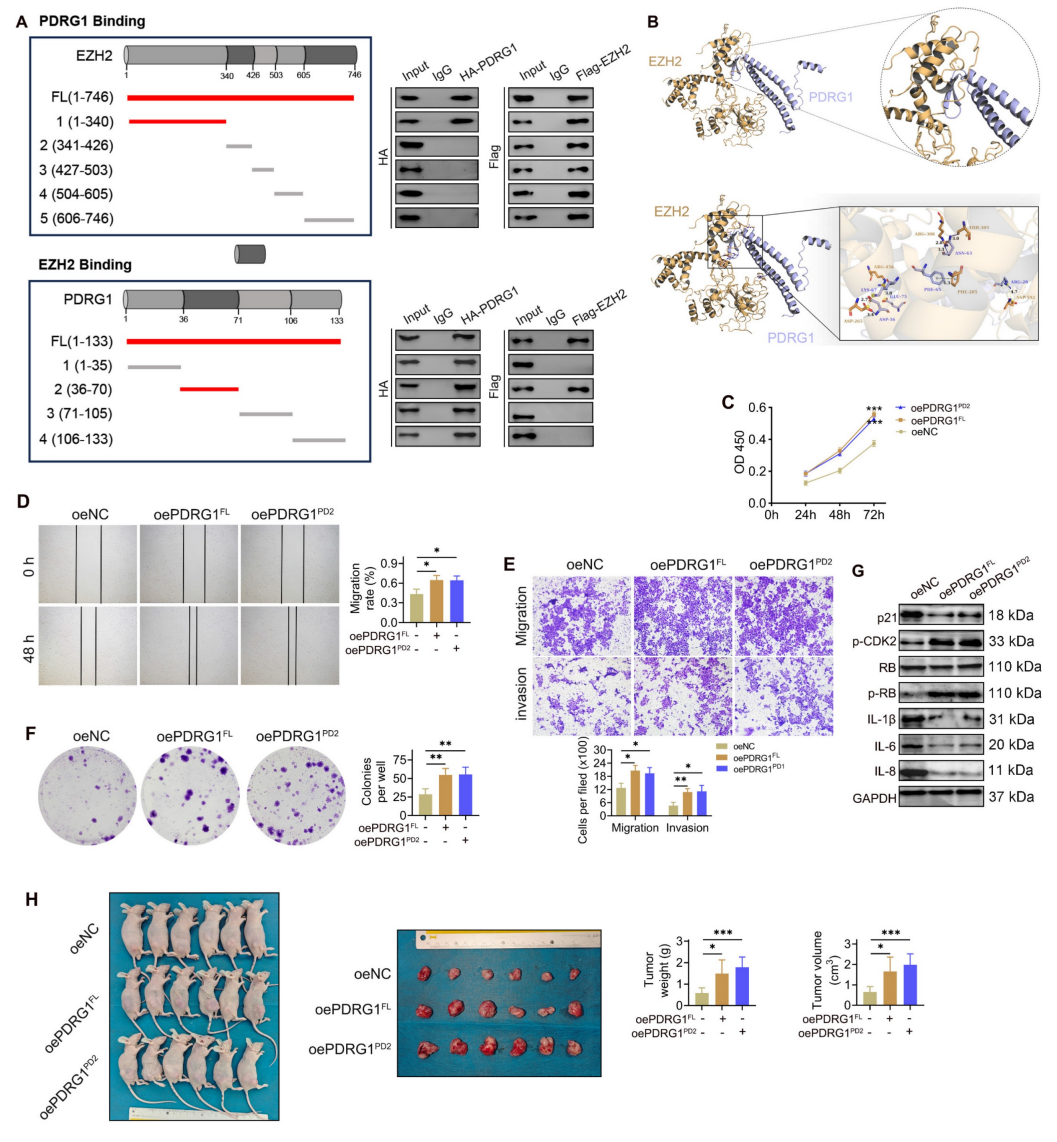
Structural identification of the PDRG1-EZH2 binding interfaces and the functional requirement of their interaction in HCC progression. (A) Co-IP using EZH2 and PDRG1 truncation mutants demonstrates that the N-terminal region of EZH2 (amino acids 1-340) and the N-terminal segment of PDRG1 (amino acids 36-70) mediate their interaction in SNU449 cells. (B) Structural modeling predicts a stable interaction interface between the N-terminal helix of PDRG1 and the N-terminal region of EZH2. (C-F) CCK-8, wound-healing, Transwell, and colony formation assays show that overexpression of the PDRG1^Δ36-70^ promotes cell proliferation, migration, invasion, and clonogenicity in SNU449 cells. All experiments were performed with three independent biological replicates (n = 3). (G) Western blot analysis indicates that the overexpression of PDRG1^Δ36-70^ suppresses p21 and SASP factors and activates p-CDK2 and p-RB in SNU449 cells. (H) Xenograft assays show that SNU449 cells expressing PDRG1^Δ36-70^ exhibit enhanced tumor growth, as indicated by increased tumor volume and tumor weight (n = 6). oePDRG1^PD2^: oePDRG1^Δ36-70^, *p < 0.05, **p < 0.01, ***p < 0.001.

To further investigate the mechanism by which PDRG1 regulates EZH2 expression, a panel of Flag-tagged EZH2 truncation mutants was co-transfected with HA-tagged full-length PDRG1 into SNU449 cells. Co-IP results showed that the N-terminal region of EZH2 (amino acids 1-340) was sufficient for binding to PDRG1, while more distal regions failed to interact (Fig. 7A, top). Conversely, the 36-70 amino acid region of PDRG1 retained robust binding to EZH2, whereas the remaining truncation mutants failed to interact (Fig. 7A, bottom), indicating that residues 36-70 constitute the critical interface mediating the PDRG1-EZH2 association. Structural modeling further corroborated these biochemical findings, revealing a stable binding interface between PDRG1 residues 36-70 and the N-terminal region of EZH2. Multiple residues were predicted to contribute to intermolecular stabilization (Fig. 7B).

To evaluate the functional significance of the PDRG1-EZH2 interaction, we generated an expression construct encoding the PDRG1 36-70 fragment (PDRG1^Δ36-70^) and compared its effects with those of full-length PDRG1 (PDRG1^FL^). CCK-8 assays showed that, similar to PDRG1^FL^, overexpression of PDRG1^Δ36-70^ promoted cell proliferation (Fig. 7C). Consistently, wound-healing and Transwell assays demonstrated that PDRG1^Δ36-70^ enhanced cell migration and invasion to a degree comparable to PDRG1^FL^ (Fig. 7D, 7E). Colony formation assays further confirmed that PDRG1^Δ36-70^ increased clonogenic growth (Fig. 7F). At the molecular level, PDRG1^Δ36-70^ suppressed p21 and SASP factors (IL-1β, IL-6, and IL-8) while elevating p-CDK2 and p-RB levels, recapitulating the regulatory profile of wild-type PDRG1 (Fig. 7G; Fig. S3). Moreover, xenograft assays showed that SNU449 cells expressing PDRG1^Δ36-70^ exhibited enhanced tumor growth, as reflected by increased tumor volume and tumor weight (Fig. 7H).

Together, these findings demonstrate that the N-terminal EZH2-binding domain of PDRG1 is indispensable for its ability to modulate EZH2 activity, suppress senescence signaling, and promote malignant phenotypes in HCC cells.

## Discussion

In this study, we identify a novel epigenetic mechanism by which PDRG1 sustains the malignant phenotype of HCC cells through repression of the cyclin-dependent kinase inhibitor p21. Our results demonstrate that PDRG1 physically associates with the histone methyltransferase EZH2, a core subunit of PRC2. Consistent with this interaction, PDRG1 modulates EZH2-dependent trimethylation of H3K27me3 at the p21 promoter, leading to transcriptional suppression of p21. Consequently, PDRG1 overexpression keeps p21 at low levels, allowing HCC cells to evade p21-induced cell senescence. Conversely, loss-of-function of PDRG1 in our experiments relieved this repression, restoring p21 expression and triggering a robust senescence program accompanied by growth arrest and diminished tumor cell proliferation. These findings place PDRG1 as an upstream regulator of the p21 checkpoint in HCC and support a previously underappreciated PDRG1-EZH2-p21 axis that contributes to tumor progression. Notably, PDRG1 is a small protein normally induced by p53 in response to DNA damage, and while it has been found in various macromolecular complexes of unclear function 29, its involvement in chromatin-based gene silencing was unknown prior to this work. Our study therefore links a DNA damage-responsive factor to Polycomb-associated repression of a key cell-cycle checkpoint, adding a new layer to the epigenetic control of senescence in HCC cells.

EZH2 is frequently overexpressed in HCC and well-known to promote oncogenesis by silencing tumor suppressors 30. In particular, EZH2-mediated repression of p21 has been documented as an important mechanism to maintain proliferation and prevent premature senescence in cancer cells 31. Our findings are aligned with this paradigm but refine it by introducing PDRG1 as a distinct upstream modulator of the EZH2-p21 pathway in HCC. Previous studies have largely focused on transcriptional or non-coding RNA regulators of EZH2 in HCC. For example, the long non-coding RNA LINC00978 was shown to bind EZH2 and recruit it to the promoters of p21 and E-cadherin, causing H3K27me3 deposition and gene silencing 32. Through this EZH2-dependent mechanism, LINC00978 suppresses p21 expression and thereby accelerates HCC cell proliferation and metastasis 32. Another study identified the DNA helicase DDX11 as an oncogenic factor that cooperates with EZH2 to repress p21: DDX11 physically interacts with EZH2 and protects it from ubiquitin-mediated degradation, resulting in sustained EZH2 activity and downregulation of p21 33. Knockdown of DDX11 in HCC cells led to upregulation of p21 and a G_1 cell cycle arrest, an effect that could be rescued by concomitant p21 silencing 33. These examples underscore that diverse oncogenic pathways in HCC converge on disabling the p21 senescence checkpoint via EZH2. However, the mechanistic novelty of our work is that it implicates a protein co-factor, PDRG1, that is intimately linked to stress and DNA damage responses 29, in shaping this epigenetic checkpoint. Unlike previously reported lncRNA scaffolds (e.g., LINC00978) or EZH2-stabilizing partners (e.g., DDX11), PDRG1 was not previously known to interface with PRC2-related repression. Thus, rather than simply reiterating EZH2 as a p21 repressor, our study adds an upstream regulatory layer by positioning PDRG1 as a molecular determinant that connects PDRG1 dysregulation to EZH2-dependent p21 suppression in HCC, thereby highlighting the unique contribution of PDRG1 to the established EZH2-p21 framework.

Our findings also highlight the central role of p21 downregulation in HCC pathogenesis and how PDRG1-EZH2 cooperation innovatively exploits this. Cellular senescence is a potent anti-tumor response, and p21 is a key effector of senescence downstream of p53 and other stresses 34, 35. In normal and premalignant hepatocytes, activation of the p53/p21 axis can induce a permanent growth arrest, preventing the clonal expansion of damaged cells 34. Indeed, recent studies demonstrate that enforcing p21 expression drives HCC cells into senescence and limits tumor growth 36. For instance, the splicing factor Prp19 has been reported to promote p21-dependent senescence in HCC cells; knockdown of Prp19 reduces p21 levels, bypasses senescence, and accelerates tumor growth *in vivo* 34. Similarly, experimental disruption of oncogenic pathways that suppress p21 (including EZH2-related networks) can trigger senescence and enhance therapeutic responses 31. In this context, the ability of PDRG1 to maintain p21 repression confers a growth advantage by disabling a major senescence inducer and cell-cycle brake.

Importantly, we also recognize the conceptual distinction between stable cellular senescence and transient cell-cycle arrest. Transient arrest is often reversible and may reflect an acute stress response, whereas stable senescence represents a more durable cell-state transition characterized by sustained checkpoint activation (frequently involving p21 and/or p16), persistent proliferative blockade, and broader senescence-associated remodeling (including secretory and chromatin features) 11. In our models, PDRG1 perturbation produced coordinated changes in p21 expression together with senescence-associated markers and growth arrest, supporting an interpretation that PDRG1 regulates a p21-linked senescence-associated checkpoint rather than merely inducing a short-lived slowdown in proliferation. Nevertheless, because arrest and senescence can exist on a spectrum depending on cellular context, we interpret our findings conservatively as evidence that PDRG1 modulates an EZH2-dependent p21 checkpoint that is closely associated with activation of a senescence program in HCC cells.

Beyond cell-autonomous growth control, senescent cells can influence tumor biology through SASP, which comprises cytokines, chemokines, growth factors, and matrix-remodeling enzymes. Therefore, the observed changes in SASP-related factors in our study may be biologically meaningful in several ways 37, 38. First, SASP can remodel the local microenvironment by altering inflammatory signaling and extracellular matrix dynamics, potentially affecting tumor cell plasticity and invasiveness. Second, SASP can modulate immune surveillance by influencing immune-cell recruitment and activation; depending on context, SASP may promote clearance of senescent cells or, conversely, establish a chronic inflammatory niche that favors tumor progression 37, 38. Accordingly, the observation that PDRG1 suppresses senescence-associated programs together with SASP-related factors suggests that PDRG1 may contribute not only to intrinsic checkpoint evasion but also to reshaping microenvironmental signaling landscapes that could support HCC progression. This perspective provides a rationale for further exploring PDRG1-associated senescence/SASP signatures as potential biomarkers and therapeutic entry points.

From a clinical and translational perspective, these discoveries regarding the PDRG1-EZH2-p21 pathway carry important implications. PDRG1 emerges as not only a mechanistic driver of HCC progression but also as a potential biomarker and therapeutic target. Consistent with our findings, PDRG1 has been found to be markedly overexpressed in HCC tissues compared to normal liver, and its high expression correlates with aggressive clinicopathological features and advanced tumor stage 39. Large-scale analyses indicate that HCC patients with elevated PDRG1 levels have significantly worse survival, underscoring PDRG1's value as a prognostic indicator 39. Importantly, PDRG1's unique role in repressing p21 and promoting escape from senescence suggests that targeting this protein could restore a fail-safe against tumor growth. In theory, therapeutic inhibition of PDRG1 (or disruption of its interaction with EZH2) would derepress p21, leading to p21 reactivation and induction of senescence or apoptosis in cancer cells. Targeting PDRG1 may offer potential advantages and complementarity relative to direct EZH2 inhibition. Because EZH2 inhibitors can broadly perturb PRC2-dependent programs, disrupting PDRG1 (or the PDRG1-EZH2 interaction) could provide a more pathway-focused means to reactivate p21 and restore senescence, and may be combined with EZH2 inhibition to enhance p21 reactivation in PDRG1-high tumors. Such an approach might synergize with existing therapies; for example, inducing senescence can improve the efficacy of chemotherapy and molecular targeted drugs in HCC 31. Moreover, PDRG1 is relatively specific in its actions upstream of p21, which raises the prospect that targeting PDRG1 could have more focused antitumor effects with potentially fewer off-target consequences than global EZH2 inhibition. Beyond therapy, PDRG1 may serve as a biomarker for patient stratification-patients with PDRG1-overexpressing tumors could be candidates for treatments that reactivate p21 or combine with epigenetic drugs. Indeed, recent literature has highlighted PDRG1 as an “important pan-cancer molecular biomarker” and a candidate for future clinical applications in cancer diagnosis and prognosis 39. Our study provides the mechanistic rationale for such applications in HCC, by showing that PDRG1 actively drives tumor progression through a defined pathway.

This study has several limitations that should be acknowledged. First, although we validated the oncogenic role of PDRG1 in two independent HCC cohorts and multiple datasets, additional multi-center cohorts with larger sample sizes are needed to further confirm its clinical prognostic value. Second, although we identified EZH2 as a key binding partner of PDRG1 and demonstrated that PDRG1 enhances EZH2-mediated H3K27me3 deposition on the p21 promoter, the precise post-translational mechanism by which PDRG1 stabilizes EZH2 protein remains to be elucidated. Third, senescence was assessed mainly through p21 and SASP-related markers; incorporating additional senescence hallmarks such as SA-β-gal staining or senescence-associated chromatin changes would further strengthen the conclusions. Finally, the downstream consequences of PDRG1-EZH2 regulation beyond p21, as well as potential cooperation with other PRC2 components, were not explored in depth. Future work addressing these unanswered questions will help refine the mechanistic understanding and translational potential of the PDRG1-EZH2-p21 axis in HCC.

## Conclusion

In summary, our work identifies PDRG1 as a clinically relevant oncogene in HCC and reveals a PDRG1-EZH2-p21 axis that suppresses cellular senescence by enhancing EZH2/H3K27me3-mediated repression of p21. These findings link PDRG1 to epigenetic control of senescence escape and support PDRG1 as a potential biomarker and therapeutic target in HCC.

## Supplementary Material

Supplementary figures and tables.

## Data Availability

The data that support the findings of this study are available on request from the corresponding author.
