# Supplementary material for: Identification of a Novel PDRG1-EZH2-p21 Pathway Controlling Senescence and Tumor Progression in Hepatocellular Carcinoma
Source: Int J Biol Sci. 2026 Feb 18;22(6):2827–41. doi: 10.7150/ijbs.129113 (PMC13050440; doi:10.7150/ijbs.129113)
Supplement: Supplementary file 1 — Supplementary figures and tables. [file ijbsv22p2827s1.pdf]

Table S1. Clinicopathologic characteristics of 86 HCC patients in our cohort.

Characteristics		Patients
		Number (%)
Age, years	≤50	35(41.18%)
	>50	50(58.82%)
Gender	Female	18(21.18%)
	Male	67(78.82%)
AFP, ng/ml	≤20	30(35.29%)
	>20	55(64.71%)
ALT, U/L	≤40	41(48.24%)
	>40	44(51.76%)
Child-pugh	A	77(90.59%)
	B	8(9.14%)
Liver cirrhosis	No	20(23.53%)
	Yes	65(76.47%)
Tumor number	Single	65(76.47%)
	Multiple	20(23.53%)
Tumor size, cm	≤5	41(48.24%)
	>5	44(51.76%)
Tumor encapsulation	Complete	54(63.53%)
	None	31(36.47%)
TNM Stage	I-II	53(62.35%)
	III-IV	32(37.65%)
Microvascular invasion	Absent	59(69.41%)
	Present	26(30.59%)
BCLC stage	A	52(61.18%)
	B + C	33(38.82%)

Table S2. Univariate and multivariate cox regression analysis of factors associated with overall survival in the TCGA-LIHC cohort

	Univariate analysis		Multivariate analysis	
Characteristics	HR(95% CI)	*P* value	HR(95% CI)	*P* value
Age				
≤ 60	Reference			
> 60	1.205 (0.850 - 1.708)	0.295		
Race				
White&Black or African American	Reference			
Asian	0.746 (0.515 - 1.080)	0.121		
Gender				
Female	Reference			
Male	0.793 (0.557 - 1.130)	0.200		
Pathologic stage				
Stage I&Stage II	Reference		Reference	
Stage III&Stage IV	2.504 (1.727 - 3.631)	**< 0.001**	2.521 (1.738 - 3.658)	**< 0.001**
AFP(ng/ml)				
≤ 400	Reference			
> 400	1.075 (0.658 - 1.759)	0.772		
Vascular invasion				
No	Reference			
Yes	1.344 (0.887 - 2.035)	0.163		
Child-Pugh grade				
A	Reference			
B&C	1.643 (0.811 - 3.330)	0.168		
Albumin(g/dl)				
< 3.5	Reference			
≥3.5	0.897 (0.549 - 1.464)	0.662		
PDRG1				
Low	Reference		Reference	
High	1.829 (1.286 - 2.602)	**< 0.001**	1.842 (1.267 - 2.677)	**0.001**
BMI				
≤ 25	Reference			
> 25	0.798 (0.550 - 1.158)	0.235		

Table S3. Univariate and multivariate cox regression analysis of factors associated with progress free interval in the TCGA-LIHC cohort

	Univariate analysis		Multivariate analysis	
Characteristics	HR(95% CI)	*P* value	HR(95% CI)	*P* value
Age				
≤ 60	Reference			
> 60	0.960 (0.718 - 1.284)	0.783		
Race				
White&Black or African American	Reference			
Asian	0.821 (0.609 - 1.107)	0.196		
Gender				
Female	Reference			
Male	0.982 (0.721 - 1.338)	0.909		
Pathologic stage				
Stage I&Stage II	Reference		Reference	
Stage III&Stage IV	2.201 (1.591 - 3.046)	**< 0.001**	1.810 (1.199 - 2.731)	**0.005**
AFP(ng/ml)				
≤ 400	Reference			
> 400	1.045 (0.698 - 1.563)	0.832		
Vascular invasion				
No	Reference		Reference	
Yes	1.676 (1.196 - 2.348)	**0.003**	1.399 (0.969 - 2.021)	0.073
Child-Pugh grade				
A	Reference			
B&C	1.395 (0.765 - 2.545)	0.277		
Albumin(g/dl)				
< 3.5	Reference			
≥ 3.5	0.911 (0.618 - 1.341)	0.636		
PDRG1				
Low	Reference		Reference	
High	1.518 (1.134 - 2.033)	**0.005**	1.448 (1.025 - 2.046)	**0.036**
BMI				
≤ 25	Reference			
> 25	0.936 (0.689 - 1.272)	0.673		

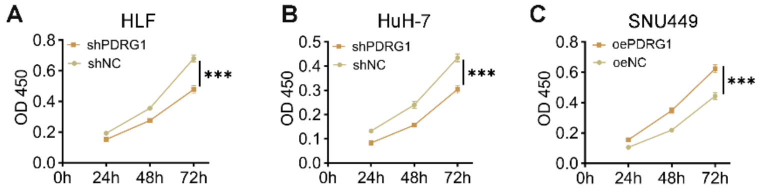

Figure S1. Additional evidence supporting the pro-proliferative role of PDRG1 in HCC cells.
(A, B) CCK-8 assays showing that PDRG1 knockdown significantly reduces cell viability in HuH-7 and HLF cells.
(C) CCK-8 assays showing that PDRG1 overexpression enhances proliferation in SNU449 cells.
***$p < 0.001$

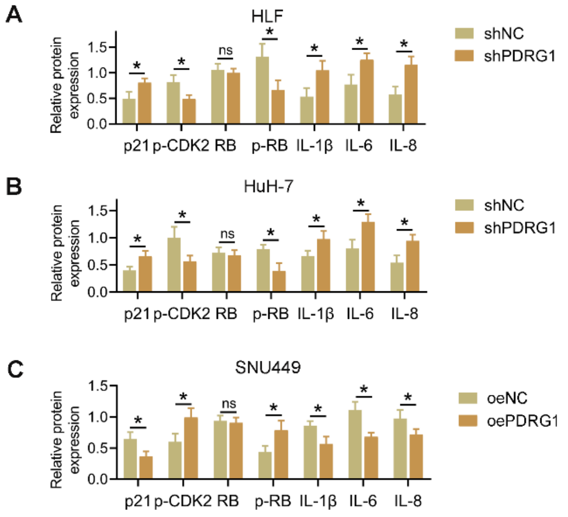

Figure S2. Quantification of p21, p-CDK2, RB, p-RB, IL-1β, IL-6, and IL-8 expression normalized to GAPDH in HLF (A), HuH-7 (B), and SNU449 (C) cells. *$p < 0.05$

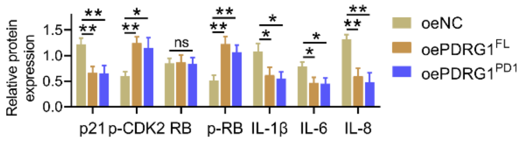

Figure S3. Quantification of protein expression showing that the overexpression of PDRG1$^{\Delta 36-70}$ suppresses p21 and SASP factors while activating p-CDK2 and p-RB. *$p < 0.05$, **$p < 0.01$
